# Catheter-Induced Cement Embolism During Attempted Ablation Procedure

**DOI:** 10.1016/j.jaccas.2021.05.005

**Published:** 2021-07-21

**Authors:** Severin Wittmer, Thomas Küffer, Christoph Gräni, Tobias Reichlin, Laurent Roten

**Affiliations:** Department of Cardiology, Inselspital, Bern University Hospital, University of Bern, Bern, Switzerland

**Keywords:** atrial fibrillation, case report, catheter intervention, cement embolism, pulmonary vein isolation, vertebroplasty, CT, computed tomography

## Abstract

Pulmonary cement embolism is a well-described complication of cement vertebroplasty (1,2). We describe the case of a patient with acute cement embolism during catheter insertion for attempted pulmonary vein isolation 1 month after cement vertebroplasty. We discuss the mechanism of acute cement embolism, possible sequelae, and treatment considerations. (**Level of Difficulty: Intermediate.**)

## Central Illustration

**Figure:**
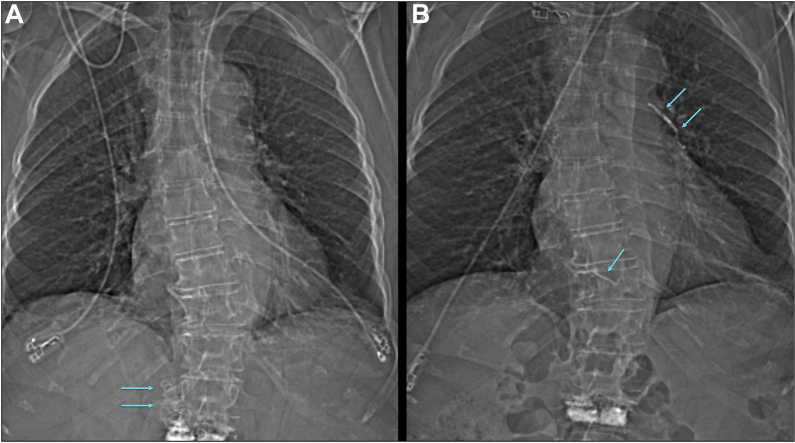

## History of Presentation

A 79-year-old woman was admitted to the hospital for redo catheter ablation of paroxysmal atrial fibrillation. On admission, she was normotensive, in sinus rhythm, and showed no signs of heart failure. A cardiac computed tomography (CT) scan showed a situs solitus with normal AV concordance, excluded intracardiac thrombi, and showed normal coronary arteries and normal cardiac anatomy. Transesophageal echocardiography revealed a mildly reduced left ventricular ejection fraction of 45%-50%, no significant valvular pathology, and no patent foramen ovale or residual shunt postablation.

**Learning Objectives**

• To recognize the possibility of cement embolism during catheter manipulation in the inferior caval vein after lumbar cement vertebroplasty.
• To ask for previous lumbar cement vertebroplasty in preprocedure screening questionnaire for procedures involving catheter manipulation in the inferior caval vein.
• To consider imaging of the inferior caval vein to rule out the presence of cement casts.

## Past Medical History

Because of symptomatic, paroxysmal atrial fibrillation and atrial flutter, the patient had undergone ablation of the cavotricuspidal isthmus 6 years before and pulmonary vein isolation and reablation of the cavotricuspidal isthmus 2 years before. Atrial fibrillation recurred after ablation and treatment with amiodarone was initiated. Due to amiodarone side effects (polyneuropathy), a redo ablation was scheduled. One month before redo ablation, the patient had a fracture of the lumbar vertebra L2 and percutaneous vertebroplasty was performed using polymethylmethacrylate cement.

## Differential Diagnosis

In view of the clinical history, no differential diagnosis was considered.

## Intervention

Anticoagulation was paused for the intervention. The patient was sedated with fentanyl, propofol, and midazolam. Access was gained via the right femoral vein, and 2 guidewires (diameter 0.032 inches) advanced blindly into the right atrium and superior caval vein, respectively. Heparin was administered, and subsequently, 2 long sheaths were placed over-the-wire into the right atrium (8.5-F steerable guiding sheath) and superior caval vein (8.5-F transseptal guiding introducer). An ablation catheter was introduced via the right atrial sheath and placed into the coronary sinus. Transseptal puncture was attempted with a Brockenbrough needle. During fluoroscopy for transseptal puncture, several large, floating foreign bodies were detected in the right atrium and right ventricle (Video 1). The procedure was aborted, and the behavior of the foreign bodies was observed. Within the next 30 minutes, they slowly migrated into the right ventricle and partly into the pulmonary arteries (Video 2).

## Investigations

**Figure 1:**
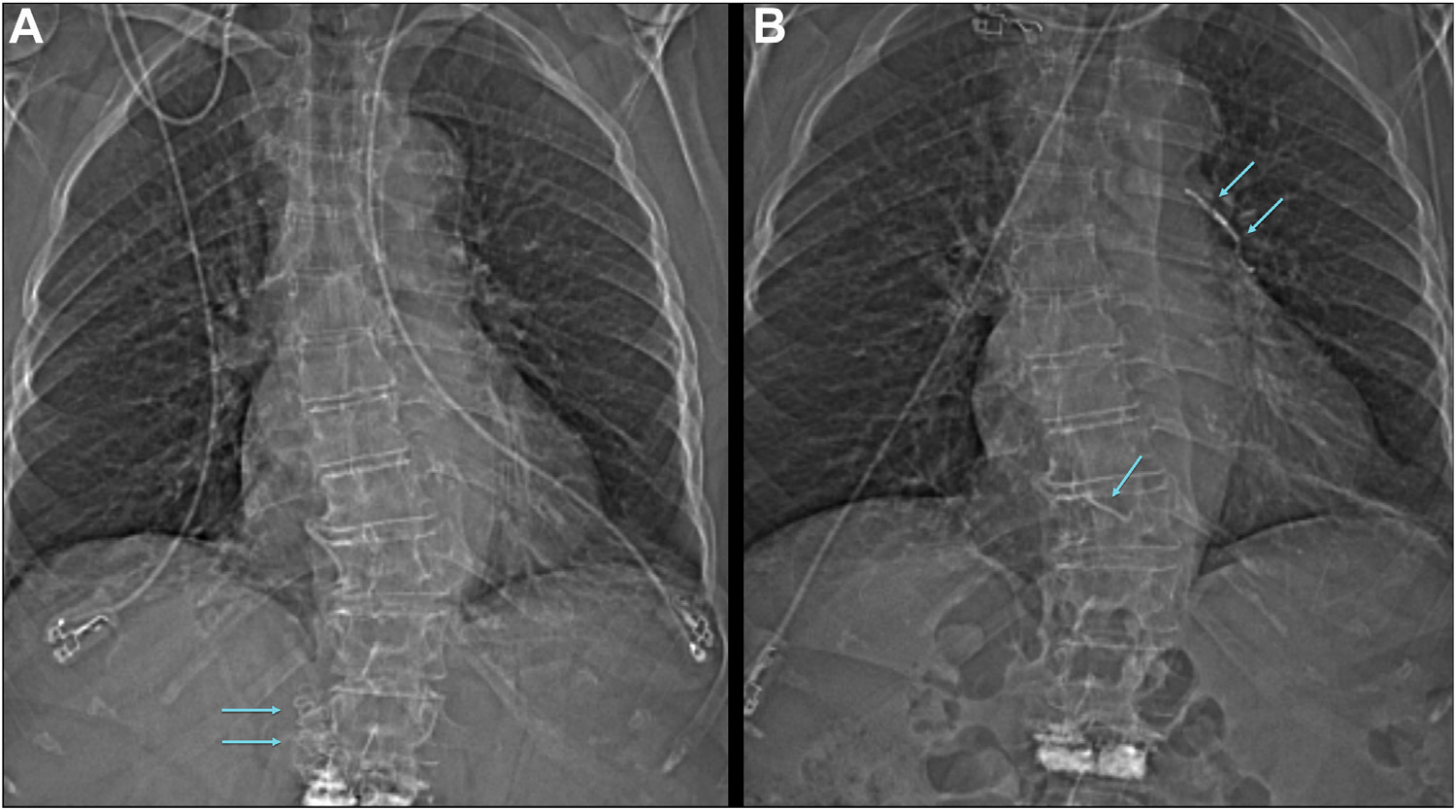
Anterior Posterior Topogram From Computed Tomography Scan **(A)** Cement casts in the inferior caval vein **(arrows)** before the ablation procedure, and **(B)** absence of cement casts in the inferior caval vein after the procedure with cement emboli **(arrows)** in the left pulmonary arteries and the right ventricle.

**Figure 2:**
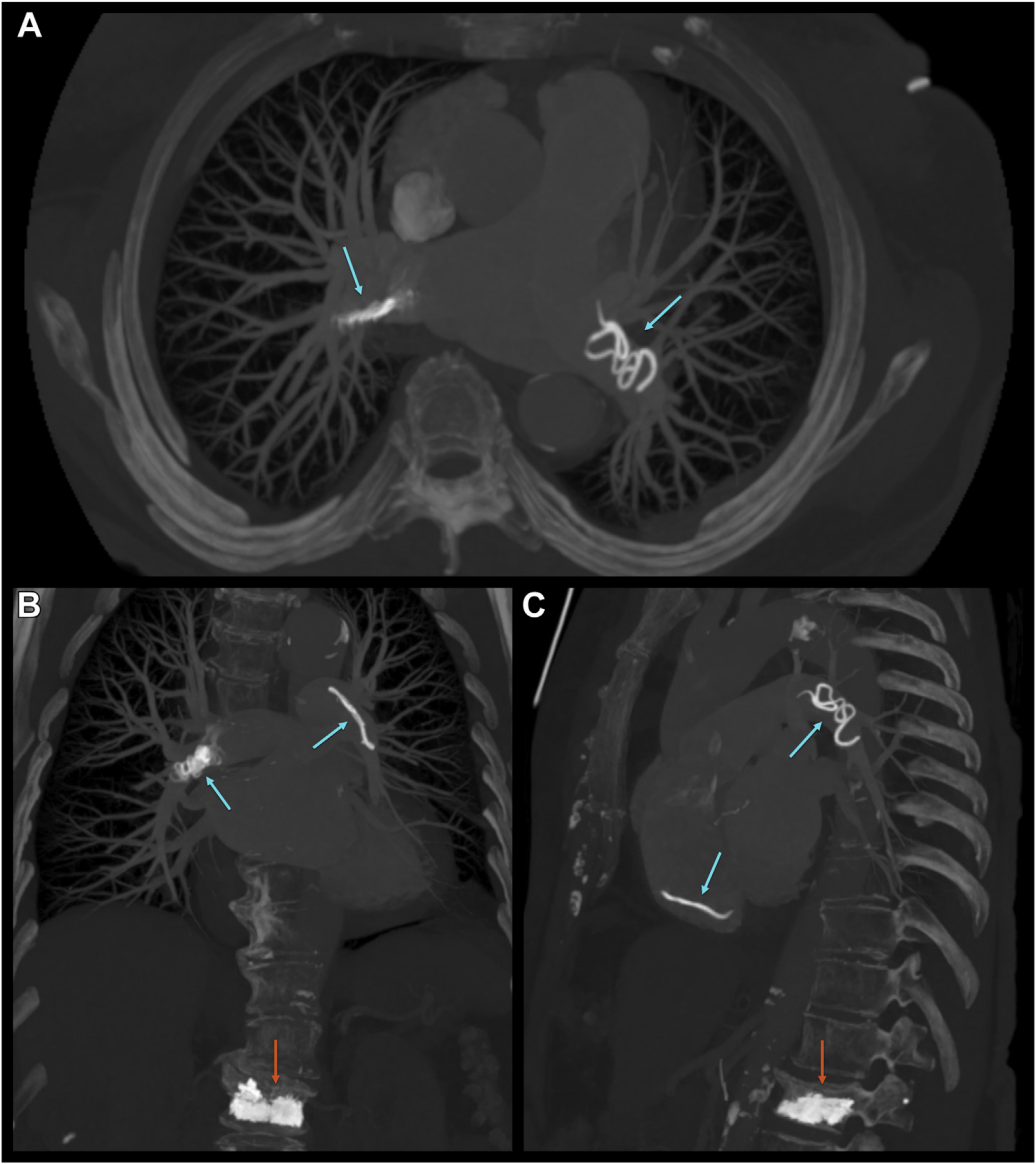
Multiplanar Maximum Intensity Projection Rendering Reconstruction Transverse **(A)**, frontal **(B)**, and sagittal view **(C)** of cement emboli **(blue arrows)** in the right ventricle, the left main pulmonary artery, and the right main pulmonary artery and cement vertebroplasty **(red arrows)**.

**Figure 3:**
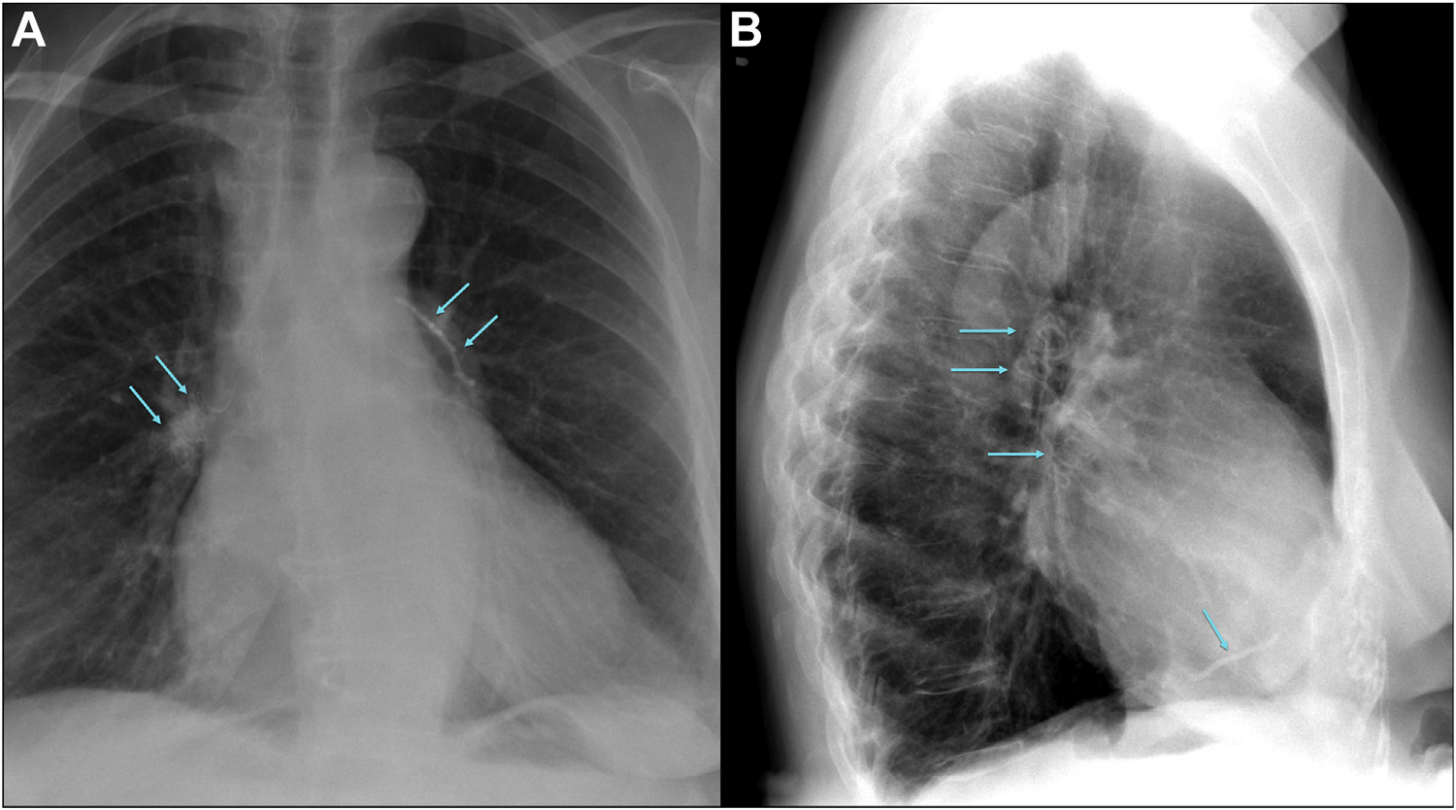
Conventional Chest X-Ray Cement emboli **(arrows)** in the pulmonary arteries and the right ventricle in posterior anterior **(A)** and lateral **(B)** view.

After abortion of the intervention, echocardiography and CT scan of the chest (Figures 1 and 2) confirmed multiple, new foreign bodies, consistent with cement embolism, in the right ventricle and pulmonary arteries. One fragment had remained in the right ventricle (5 × 45 mm). Two others were located in the pulmonary arteries: 1 in the main right artery (8 × 27 mm) and 1 in the main left artery (15 × 37 mm). Pericardial and pleural effusion were ruled out, and no signs of pulmonary artery obstruction or pulmonary thrombus were detected. The function of the tricuspid and pulmonary valves was normal, and no significant pressure gradient was measurable across the tricuspid valve. A chest radiography on the following day showed unchanged findings (Figure 3).

## Management

After diagnosis of acute cement embolism, full anticoagulation with heparin was continued, and the patient was admitted to the intermediate care unit for surveillance. The patient remained asymptomatic and hemodynamically stable with normal oxygen saturation, and was re-anticoagulated with rivaroxaban before discharge.

## Discussion

Percutaneous vertebroplasty has been a common procedure for the treatment of osteoporotic vertebral fracture for many years (3,4). Asymptomatic cement leakage outside of the vertebral body is observed in up to 81% of all procedures (5). The reason for cement leakage is application of inappropriate cement volume, viscosity, or injection pressure (6). The clinical significance of cement leakage is the subject of debate (1,5,7). In some cases, cement can leak into the venous system and subsequently cause pulmonary embolism. The reported incidence of pulmonary embolism ranges from 3.5%-23%, depending on the method of analysis (2,8). Cases of severe and even fatal outcome of cement embolism causing, eg, cardiac perforation, tricuspid valve dysfunction, or pulmonary artery obstruction, have been described (9, 10, 11).

Data regarding treatment of cement embolism are limited (12). Krueger et al. (6) suggest anticoagulation in symptomatic patients and patients with central pulmonary embolism to prevent thrombus formation on embolized fragments. However, no general recommendation for anticoagulation is issued for asymptomatic patients (6). Open surgical or percutaneous intervention for cement extraction has been described in case reports and may be considered in hemodynamically compromised or symptomatic patients (13,14). A treatment decision tree on when to perform surgery is provided in the paper by Barakat et al. (15).

Considering patient history, imaging evidence, and acute migration of foreign bodies during the procedure, the most likely explanation in our case is acute embolization of cement casts residing in the inferior caval vein caused by catheter manipulation. Oral anticoagulation was indicated because of atrial fibrillation, and no additional therapy was required.

## Follow-Up

Three months after the intervention, the patient was doing well without signs of pulmonary obstruction. Treatment with amiodarone was reinitiated, and no further ablation procedure was planned because of the anticipated risk of repeat cement embolism. Implantation of a conventional cardiac pacemaker with atrioventricular node ablation via a jugular vein approach was discussed with the patient as an alternative to amiodarone treatment.

## Conclusions

To our knowledge, this is the first case report of acute cement embolism caused by catheter manipulation in the inferior caval vein. After cement vertebroplasty, pre-interventional imaging (such as vascular ultrasound or abdominal CT scan) should be considered prior to procedures requiring access via the inferior caval vein, to exclude cement casts in the venous system.

## Funding Support and Author Disclosures

The authors have reported that they have no relationships relevant to the contents of this paper to disclose.

## Appendix

Supplemental Video 1 Fluoroscopy. Cement fragments floating intracardially in left lateral view.

Supplemental Video 2 Fluoroscopy. Cement fragments floating intracardially and in the left pulmonary artery in anterior posterior view.

## References

[1] Hsieh M.K., Kao F.C., Chiu P.Y. (2019). Risk factors of neurological deficit and pulmonary cement embolism after percutaneous vertebroplasty. J Orthop Surg Res.

[2] Kim Y.J., Lee J.W., Park K.W. (2009). Pulmonary cement embolism after percutaneous vertebroplasty in osteoporotic vertebral compression fractures: incidence, characteristics, and risk factors. Radiology.

[3] Yuan W.H., Hsu H.C., Lai K.L. (2016). Vertebroplasty and balloon kyphoplasty versus conservative treatment for osteoporotic vertebral compression fractures: a meta-analysis. Medicine (Baltimore).

[4] Chandra R.V., Maingard J., Asadi H. (2018). Vertebroplasty and kyphoplasty for osteoporotic vertebral fractures: what are the latest data?. AJNR Am J Neuroradiol.

[5] Schmidt R., Cakir B., Mattes T., Wegener M., Puhl W., Richter M. (2005). Cement leakage during vertebroplasty: an underestimated problem?. Eur Spine J.

[6] Krueger A., Bliemel C., Zettl R., Ruchholtz S. (2009). Management of pulmonary cement embolism after percutaneous vertebroplasty and kyphoplasty: a systematic review of the literature. Eur Spine J.

[7] Lee M.J., Dumonski M., Cahill P., Stanley T., Park D., Singh K. (2009). Percutaneous treatment of vertebral compression fractures: a meta-analysis of complications. Spine (Phila Pa 1976).

[8] Anselmetti G.C., Corgnier A., Debernardi F., Regge D. (2005). Treatment of painful compression vertebral fractures with vertebroplasty: results and complications. Radiol Med.

[9] Caynak B., Onan B., Sagbas E., Duran C., Akpinar B. (2009). Cardiac tamponade and pulmonary embolism as a complication of percutaneous vertebroplasty. Ann Thorac Surg.

[10] Son K.H., Chung J.H., Sun K., Son H.S. (2008). Cardiac perforation and tricuspid regurgitation as a complication of percutaneous vertebroplasty. Eur J Cardiothorac Surg.

[11] Yoo K.Y., Jeong S.W., Yoon W., Lee J. (2004). Acute respiratory distress syndrome associated with pulmonary cement embolism following percutaneous vertebroplasty with polymethylmethacrylate. Spine (Phila Pa 1976).

[12] Geraci G., Lo Iacono G., Lo Nigro C., Cannizzaro F., Cajozzo M., Modica G. (2013). Asymptomatic bone cement pulmonary embolism after vertebroplasty: case report and literature review. Case Rep Surg.

[13] Tozzi P., Abdelmoumene Y., Corno A.F., Gersbach P.A., Hoogewoud H.M., von Segesser L.K. (2002). Management of pulmonary embolism during acrylic vertebroplasty. Ann Thorac Surg.

[14] Bose R., Choi J.W. (2010). Successful percutaneous retrieval of methyl methacrylate orthopedic cement embolism from the pulmonary artery. Catheter Cardiovasc Interv.

[15] Barakat A.S., Owais T., Alhashash M. (2018). Presentation and management of symptomatic central bone cement embolization. Eur Spine J.

